# Comparative genomic analysis of Shiga toxin-producing and non-Shiga toxin-producing *Escherichia coli* O157 isolated from outbreaks in Korea

**DOI:** 10.1186/s13099-017-0156-2

**Published:** 2017-02-06

**Authors:** Taesoo Kwon, Won Kim, Seung-Hak Cho

**Affiliations:** 1 Interdisciplinary Program in Bioinformatics, Seoul National University, 1 Gwanak-ro, Gwanak-gu, Seoul, 151-742 Republic of Korea; 2 Division of Biosafety Evaluation and Control, Korea National Institute of Health, Cheongju, 363-951 Republic of Korea; 3 Division of Enteric Diseases, Center for Infectious Diseases, Korea National Institute of Health, Cheongju, 363-951 Republic of Korea

**Keywords:** Shiga-like toxin-producing *Escherichia coli* O157, Non-Shiga-like toxin-producing *Escherichia coli* O157, Draft genome, Comparative genomics, Alpha-hemolysin

## Abstract

**Background:**

The Shiga toxin-producing *Escherichia coli* (STEC) O157 strain NCCP15739 and non-STEC O157 strain NCCP15738 were isolated from outbreaks in Korea. We characterized NCCP15739 and NCCP15738 by genome sequencing and a comparative genomic analysis using two additional strains, *E. coli* K-12 substr. MG1655 and O157:H7 EDL933.

**Results:**

Using the Illumina HiSeq 2000 platform and the RAST server, the whole genomes of NCCP15739 and NCCP15738 were obtained and annotated. NCCP15739 and NCCP15738 clustered with different *E. coli* strains based on a whole-genome phylogeny and multi-locus sequence typing analysis. Functional annotation clustering indicated enrichment for virulence plasmid and hemolysis-related genes in NCCP15739 and conjugation- and flagellum-related genes in NCCP15738. Defense mechanism- and pathogenicity-related pathways were enriched in NCCP15739 and pathways related to the assimilation of energy sources were enriched in NCCP15738. We identified 66 and 18 virulence factors from the NCCP15739 and NCCP15738 genome, respectively. Five and eight antibiotic resistance genes were identified in the NCCP15739 and NCCP15738 genomes, respectively. Based on a comparative analysis of phage-associated regions, NCCP15739 and NCCP15738 had specific prophages. The prophages in NCCP15739 carried virulence factors, but those in NCCP15738 did not, and no antibiotic resistance genes were found in the phage-associated regions.

**Conclusions:**

Our whole-genome sequencing and comparative genomic analysis revealed that NCCP15739 and NCCP15738 have specific genes and pathways. NCCP15739 had more genes (410), virulence factors (48), and phage-related regions (11) than NCCP15738. However, NCCP15738 had three more antibiotic resistance genes than NCCP15739. These differences may explain differences in pathogenicity and biological characteristics.

**Electronic supplementary material:**

The online version of this article (doi:10.1186/s13099-017-0156-2) contains supplementary material, which is available to authorized users.

## Background

In 1983, outbreaks of EHEC O157:H7 in humans were first reported [1–3]. Since then, EHEC has been recognized as an important food-borne pathogen that causes hemorrhagic colitis and hemolytic uremic syndrome [4, 5]. Shiga toxin (Stx) is the major virulence factor and a defining characteristic of EHEC. Shiga toxin-producing *Escherichia coli* (STEC) strains produce one or two major Shiga toxins, designated Stx1 and Stx2 [4]. Typical STEC strains possess a 35-kb locus of enterocyte effacement (LEE) pathogenicity island containing *eae* [6], which encodes an outer membrane protein (intimin) required for intimate attachment to epithelial cells; this pathogenicity island is also found in EPEC strains. LEE encodes a type III secretion system (TTSS) through which *E. coli* secretes proteins, resulting in the delivery of effector molecules to the host cell and disrupting the host cytoskeleton [7–10]. STEC strains cause hemolytic-uremic syndrome and hemorrhagic colitis.

Numerous comparative genomics studies of STEC O157 and non-O157 STEC have been performed, but non-STEC O157 has not been a focus of past research. Few cases of non-STEC O157 have been reported in human patients with diarrhea [11]. Moreover, there are no whole-genome sequencing data or comparative genomics studies of non-STEC O157 strains. However, there was a recent outbreak of non-STEC O157 in human hosts in Korea [12]. Even though non-STEC O157 does not produce Shiga-like toxins, it could be a public health problem because it is pathogenic and causes diarrhea in humans. STEC NCCP15739 [13] and non-STEC NCCP15738 [12] were isolated from the feces of two Korean patients with diarrhea. To characterize NCCP15739 and NCCP15738 as well as the origin of pathogenicity, whole-genome sequencing and comparative genomic analyses using two additional strains, *E. coli* K-12 substr. MG1655 and O157:H7 EDL933 (as non-STEC and STEC reference strains, respectively), were performed.

## Methods

### Strain, isolation, and serotyping

*Escherichia coli* were isolated from patients with diarrhea using MacConkey agar and Trypticase Soy Broth containing vancomycin (Sigma Co., St. Louis, MO, USA). Candidate colonies were identified based on phenotypes and biochemical properties using the API20E system (Biomerieux, Marcy l’Etoile, France). The O antigen of the isolates was determined using the methods of Guinee et al. [14] with all available O (O1 to O181) antisera (Lugo, Spain, http://www.lugo.usc.es/ecoli). The isolated strains have been deposited in the National Culture Collection for Pathogens (NCCP) at the Korea National Institute of Health under accession numbers NCCP15739 [13] and NCCP15738 [12]. *E. coli* K-12 substr. MG1655 and NCCP15738 were used as reference strains for non-STEC and EHEC O157:H7 str. EDL933 was used as the reference strain for STEC.

### Library preparation and whole genome sequencing

The Illumina HiSeq 2000 platform was used for the whole genome sequencing of NCCP15739 and NCCP15738 (Theragen Etex Bio Institute, Suwon, Republic of Korea).

### Genome assembly and annotation

A de novo assembly was performed using SOAPdenovo (version 1.05) [15]. Only scaffolds longer than 500 bp were used for further analysis. Annotated open reading frames of the NCCP15739 and NCCP15738 genomes were identified using the RAST (Rapid Annotation using Subsystem Technology, version 4.0) [16] server. The genomes of two reference strains, K-12 substr. MG1655 and O157:H7 str. EDL933, were re-annotated using the RAST server. For the comparison of the coding sequences (CDSs) of the four strains, OrthoMCL (version 2.0.9) was used [17]. The sequence similarity and coverage [18] were considered simultaneously to assess the orthologous proteins of all four *E. coli* strains.

Functional annotation enrichment in the set of genes in NCCP15739 and NCCP15738 was performed using the Database for Annotation, Visualization and Integrated Discovery (DAVID) (http://david.abcc.ncifcrf.gov). To identify lineage-specific genes in the NCCP15739 and NCCP15738 genomes, the BLAST Score Ratio (BSR) was calculated. Unique genes with a BSR score of ≤0.4 were selected. A comparative KEGG metabolic pathway analysis was conducted for the total CDSs of NCCP15739 and NCCP15738 using Model SEED (version 1.0). To investigate the virulence factor genes in the four *E. coli* strains, a BLAST search of the total ORFs of the four *E. coli* strains against the virulence factor genes of *E. coli* listed in VFDB [19] was performed with an e-value threshold of 1e-5. We determined the antibiotic resistance genes in the genome sequences of the four *E. coli* strains using ResFinder 2.1 (https://cge.cbs.dtu.dk/services/ResFinder/) [20]. To compare the genomic structures among the four strains, the genomic scaffolds of NCCP15739, NCCP15738, *E. coli* K-12 substr. MG1655, and O157:H7 EDL933 were aligned using the progressive alignment algorithm of Mauve (version 2.3.1) [21]. After the alignment, the scaffolds of NCCP15739 were reordered against the complete genome of *E. coli* O157:H7 EDL933 using the Move Contig tool of Mauve. The scaffolds of NCCP15738 were reordered against the genome sequence of *E. coli* K-12 substr. MG1655. The BLAST algorithm was used to identify syntenic genes and to analyze the genes of interest. The resulting reordered scaffolds and syntenic genes were visualized using Circos (version 0.64) [22].

### Phylogenetic analysis

To calculate the evolutionary distances among 44 *E. coli*, including NCCP15739 and NCCP15738, concatenated whole genomes and multi-locus sequence typing (MLST) genes [23, 24] were used. Three *Shigella* genome sequences were included in the phylogenetic analysis as an outgroup. The seven MLST genes were *adk*, *fumC*, *gyrB*, *icd*, *mdh*, *purA*, and *recA* from 44 *E. coli* strains according to the protocol described in the *E. coli* MLST database (http://mlst.warwick.ac.uk/mlst/dbs/Ecoli/documents/primersColi_html) [25]. Any locus with a gap or indel was excluded from the analysis [26]. Multiple sequence alignments of the whole genomes and MLST genes were obtained using Mugsy (version 1.2.3) [27]. The generalized time-reversible [28] + CAT model [29] was employed to infer the approximately maximum-likelihood phylogenetic trees with 1000 iterations using FastTree (version 2.1.7) [30]. FigTree (version 1.3.1) (http://tree.bio.ed.ac.uk/software/figtree/) was used for tree visualization.

### Analysis of mobile genetic elements

To identify insertion sequences (ISs), all ISs were downloaded from the IS Finder DB (http://www-is.biotoul.fr), and the genome sequences of four *E. coli* strains, NCCP15739, NCCP15738, K-12 substr. MG1655, and EDL933, were mapped to the sequence database using RepeatMasker (version 4.0.1) (http://www.repeatmasker.org). Phage-associated regions in the genome sequences of the four *E. coli* strains were predicted using the PHAST server [31]. Genomic scaffolds, including prophages, were confirmed based on the RAST annotation results.

### Quality assurance

The genomic DNAs were purified from a pure culture of a single bacterial isolate of NCCP15739 and NCCP15738. Potential contamination of the genomic libraries by other microorganisms was evaluated using a BLAST search against the non-redundant database.

## Results and discussion

### General features

**Fig. 1:**
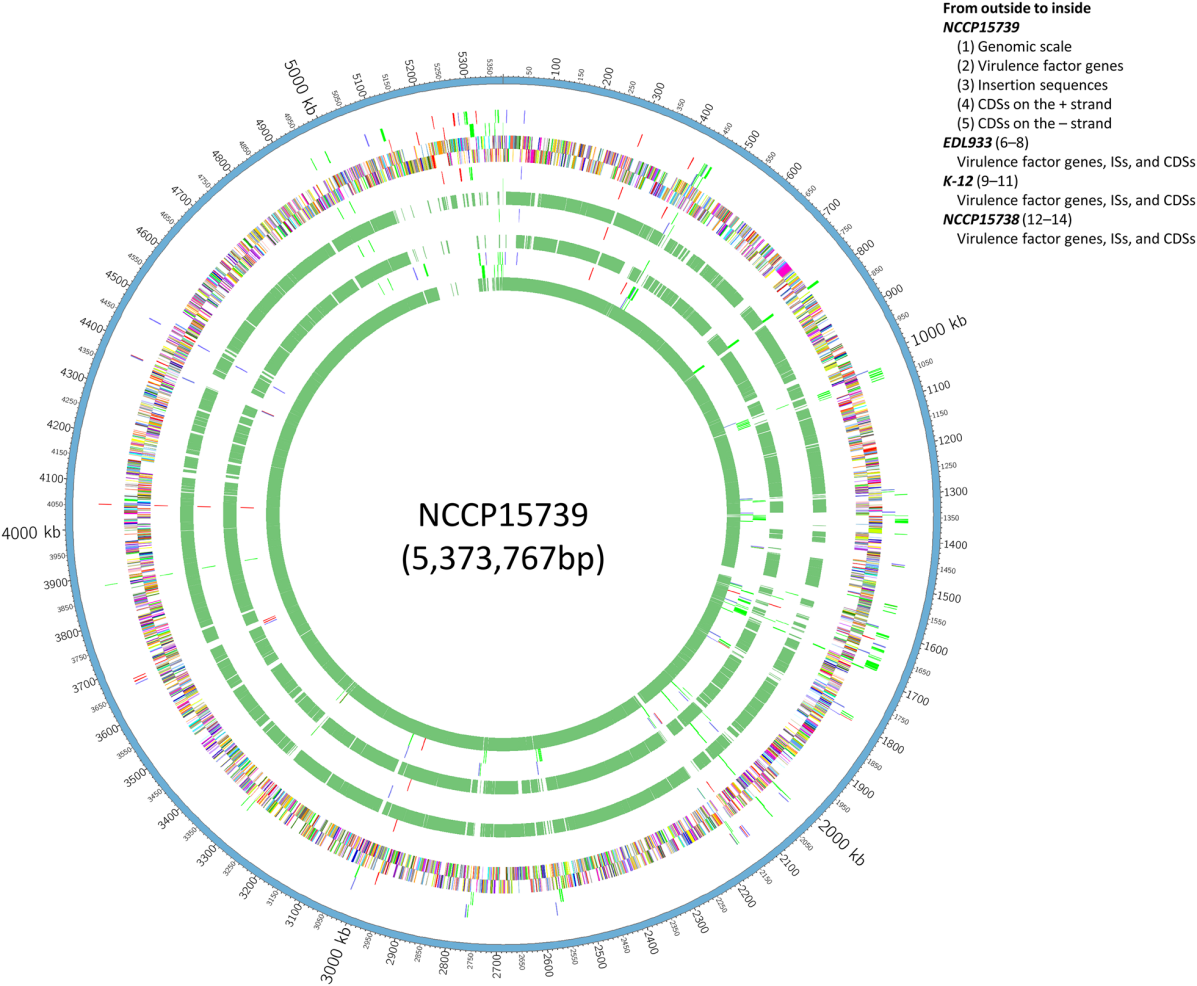
Circular map of the NCCP15739 and NCCP15738 draft genomes. Circular map of genes and genome statistics were visualized for NCCP15739 and NCCP15738 using Circos (version 0.64). All CDSs are syntenic regions of NCCP15739 that were determined using BLAST searches

The draft genome size of NCCP15739 was 5,373,767 bp and NCCP15738 was 5,005,278 bp. The G+C contents of NCCP15739 and NCCP15738 were 50.25 and 50.65%, respectively. The genomic features of *E. coli* strains used in the analysis, including NCCP15739 and NCCP15738, are summarized in Table 1. Based on a RAST analysis, 5190 putative CDSs from NCCP15739 and 4780 putative CDSs from NCCP15738 (Fig. 1; Additional file 1: Table S1) were identified. The syntenic regions between NCCP15739 and three other *E. coli* strains based on a BLAST search are depicted on the reordered contigs of NCCP15739 in Fig. 1.

**Table 1:** Genomic features of *Escherichia coli* strains used in this study

Strain	NCCP15739	NCCP15738	*E. coli* str. K-12 substr. MG1655	*E. coli* O157:H7 str. EDL933
Genome (Mb)	5.37	5.0	4.64	5.62
%GC	50.25	50.65	50.8	50.4
Total open reading frames	5226	4811	4527	5411
tRNAs	30	27	88	98
rRNAs	6	4	22	87

### Phylogenetic analysis

**Fig. 2:**
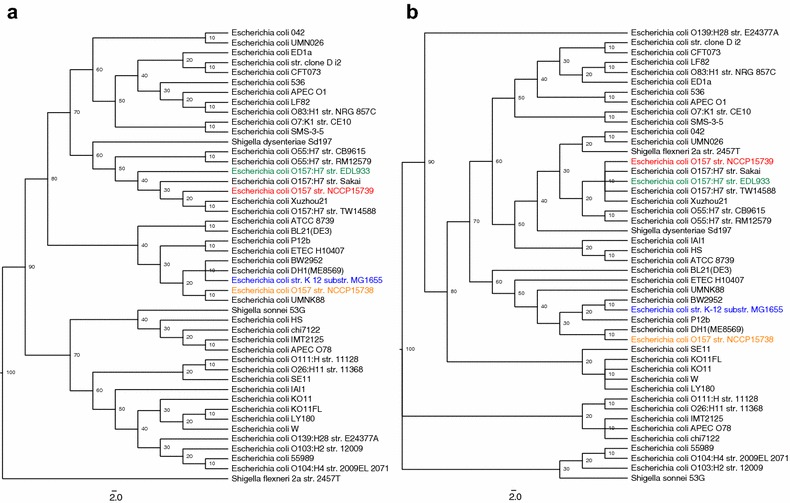
Phylogenetic tree of NCCP15739 and NCCP15738. **a** Whole-genome phylogeny, **b** multi-locus sequence typing phylogeny. Evolutionary time was scaled by 100; lower values imply a relatively recent branching event. The *scale* indicates the number of substitutions per site. NCCP15739, NCCP15738, and the reference strains are highlighted in *different colors*: NCCP15739 (*red*), NCCP15738 (*orange*), *Escherichia coli* K-12 substr. MG1655 (*blue*), and *E. coli* O157:H7 str. EDL933 (*green*)

A whole-genome phylogenetic analysis of 44 *E. coli* strains revealed that NCCP15739 is closely related to the pathogenic *E. coli* Xuzhou21 and TW14588 (Fig. 2a). However, a multilocus sequence analysis showed that NCCP15739 is closely related to O157:H7 serotypes, such as *E. coli* O157:H7 Sakai, EDL933, TW14588, and *E. coli* Xuzhou21 (Fig. 2b). The serotype O157:H7 clustered into a recently diverged group according to the MLST-based phylogeny. Based on the whole-genome phylogenetic analysis, NCCP15738 was grouped with UMNK88 (Fig. 2a), but it grouped with DH1 (ME8569) based on MLST analyses (Fig. 2b). The clusters in the whole-genome phylogenetic tree and the MLST phylogenetic tree were different; we think the difference comes from how many genotypes were considered in the phylogenetic analysis. The whole-genome phylogenetic tree considered all of variation throughout the whole-genome, but MLST phylogenetic tree only considered the genotypes of the seven MLST genes. Based on the phylogenetic analysis, we concluded that NCCP15739 and NCCP15738 are different strains belonging to their own groups.

### Functional annotation clustering

Based on BSR scores, we selected 534 genes from NCCP15739 and 651 genes from NCCP15738 for functional annotation clustering (Additional file 1: Table S1). According to this analysis, 534 genes of NCCP5739 were classified into 7 groups and 651 genes of NCCP15738 were classified into 8 groups. In NCCP15739, the virulence plasmid and hemolysis-related genes were enriched, while the NCCP15738 genome exhibited enrichment for conjugation- and flagellum-related genes (Table 2). In particular, the flagellum is an important characteristic of NCCP15738 because the strain has a dual flagellar system [12], like those found in *Vibrio parahaemolyticus*, *Aeromonas* spp., and *Rhodospirillum centenum* [32]. NCCP15738 had 65 genes encoding flagellar biosynthesis or structural proteins.

**Table 2:** Functional annotation clustering using the DAVID (http://david.abcc.ncifcrf.gov)

Cluster	Strain	Term	Count	Enrichment score	P value	Benjamini
1	NCCP15738	Conjugation	6	4.03	0.000	0.000
	NCCP15739	Virulence plasmid of *Escherichia coli* O157:H7	40	50.4	0.000	0.000
2	NCCP15738	Flagellum	5	2.76	0.000	0.005
	NCCP15739	Hemolysis	4	5.9	0.000	0.000
3	NCCP15738	Viral capsid assembly	3	1.941	0.004	0.180
	NCCP15739	Bacterial secretion system	6	4.36	0.000	0.000
4	NCCP15738	Ribonucleotide binding	9	1.043	0.033	0.674
	NCCP15739	Methylation	4	2.77	0.003	0.021
5	NCCP15738	Copper ion binding	3	0.521	0.010	0.505
	NCCP15739	Transfer region of pO113 from enterohemorrhagic *Escherichia coli*	5	1.76	0.000	0.004
6	NCCP15738	DNA recombination	3	0.496	0.029	0.336
	NCCP15739	Metal ion binding	4	0.314	0.471	1.000
7	NCCP15738	Two-component signal transduction system (phosphorelay)	4	0.423	0.070	0.687
	NCCP15739	Transmembrane protein	4	0.198	0.280	0.927

### Metabolic pathway comparison

Based on a metabolic pathway comparison, we found that seven pathways were more developed in NCCP15739 than in NCCP15738. Genes in the pathways that determine folate biosynthesis, purine metabolism, amino sugar metabolism, atrazine degradation [33], urea cycle, amino acid metabolism, and the biosynthesis of siderophores [34–36] were more highly enriched in NCCP15739. For example, the folate biosynthesis pathway had more genes in NCCP15739 than in NCCP15738 (Additional file 2: Table S2). Folate is important for frequent divisions and rapid cell growth because it is required for methylation reactions and nucleic acid synthesis [37]. The pathways enriched in NCCP15739 were closely related to defense mechanisms and the pathogenicity of bacteria. NCCP15739 is pathogenic and causes hemolytic-uremic syndrome in the host [13].

By contrast, sixteen pathways were more developed in NCCP15738. The enriched pathways in NCCP15738 were responsible for the assimilation of various energy sources (Additional file 2: Table S2). Genes in the pathways that determine tyrosine metabolism, pentose and glucuronate interconversion [38], phenylalanine metabolism [39], galactose metabolism [40], glycerolipid metabolism, and ascorbate and aldarate metabolism were more highly enriched in NCCP15738. A comparative genomic analysis with the reference strains *E. coli* K-12 substr. MG1655 and O157:H7 EDL933 showed that NCCP15738 has a dual flagellar system [12]. However, we did not observe its locomotion and did not test its function in the strain; the structure and function should be investigated in further studies.

### Virulence factors

We detected 66 and 18 [12] virulence factors from NCCP15739 and NCCP15738, respectively (Additional file 3: Table S3). All 18 virulence genes of NCCP15738 were shared with NCCP15739; NCCP15738 did not contain any unique virulence factors. The 66 virulence genes of NCCP15739 were grouped into 7 categories: adherence, autotransporter, iron uptake, LEE-encoded TTSS effectors, non-LEE-encoded TTSS effectors, secretion system, and toxins. Some virulence factors were found in NCCP15739, but not in NCCP1738, i.e., genes in the adherence category (*eae*, *paa*, and *toxB*), autotransporter category [the aida (adhesion involved in diffuse adherence)-related genes *espP* and *sat*), iron uptake category (hemin uptake-related genes (*chuA*, *S*, *T*, *U*, *W*, *X*, and *Y*), salmochelin and siderophore-related genes (*iroB*, *D*, and *N*)], toxins [alpha-hemolysin-related genes (*hlyA*, *B*, *C*, and *D*), and Shiga toxin-related genes (*stx1A*, *1B*, *2A*, and *2B*)]. Notably, in the non-LEE and LEE-encoded TTSS effector category, *espG*, *map*, *tir*, *espJ*, *nleA*/*espI*, and *nleC* were found in NCCP15739. Many LEE TTSS-related genes (*cesD2*, *F*, *T*, *escC*, *D*, *F*, *J*, *N*, *R*, *S*, *T*, *U*, *V*, *espA*, *B*, *D*, *glrR*, *ler*, *sepL,* and *Q*) belonged to the secretion systems category. NCCP15739 possessed all of the TTSS effectors and secretion system-related genes. However, NCCP15738 did not have all LEE TTSS-related genes, and it harbored only one secretion gene, *escR* [41], which might be lineage-specific (percent sequence identity = 46.85%). In the toxin category, alpha-hemolysin was a main virulence gene in STEC strains. The alpha-hemolysin–related genes (*hlyA*, *B*, *C*, and *D*) were only present in NCCP15739. It is thought to be acquired by horizontal gene transfer via conjugative plasmids [42]. The 92-kb virulence plasmid pO157 carried 3.4 kb of hemolysin genes [43]. In the NCCP15739 genome, pO157 was on scaffolds 35 and 38. Shiga toxin-related genes (*stx1A*, *1B*, *2A*, and *2B*) [44] were present in NCCP15739, but no toxin genes were found in NCCP15738. In brief, the NCCP15738 strain had fewer virulence factors than NCCP15739. However, NCCP15738 is pathogenic and causes diarrhea in human hosts. We propose that the strain NCCP15738 is a model organism for studies of pathogenicity in non-STEC O157 strains because its genome does not contain toxin-related genes. To identify the virulence genes related to diarrhea in humans, additional studies are needed.

### Phage-associated regions

Prophages are mobile genetic elements that deliver antimicrobial-resistance genes [45] or virulence factors [46] to bacterial hosts and contribute to the diversity of host genomes [47]. We identified sixteen phage-associated regions (S1–S16) from the NCCP15739 genome and five phage-associated regions (N1–N5) from the NCCP15738 genome using the PHAST algorithm (Additional file 4: Table S4). Only five of the sixteen phages in NCCP15739 were intact, whereas all five phages in NCCP15738 were intact. Based on a BLAST search, only one phage-associated region, i.e., the N3 region from NCCP15738, was identical to the S2 region from the NCCP15739 genome, whereas the four remaining phages (N1, N2, N4, and N5) were specific to NCCP15738. In terms of virulence, the S2, S4, S11, S12, and S13 regions in NCCP15739 had the virulence factors *nleC*, *stx2A* and *stx2B*, *paa*, *nleA/espI*, *espJ* and *stx1A,* and *stx1B*, respectively. Meanwhile, NCCP15738 had no virulence factors in the phage-associated regions. Therefore, we hypothesized that prophages are not causal factors of virulence in NCCP15738. In addition, we examined antibiotic resistance-related genes in prophage regions of NCCP15739 and NCCP15738, but no antibiotic resistance genes were found in either genome (Additional file 5: Table S5). According to these results, we concluded that prophages are not vehicles of antibiotic resistance genes in NCCP15739 and NCCP15738.

### Future directions

STEC O157 NCCP15739 and non-STEC NCCP15738 belong to the O157 serotype, which has strong pathogenicity and can cause foodborne disease. In this study, we performed a comparative genomic analysis of NCCP15739, NCCP15738, *E. coli* K-12 substr. MG1655, and O157:H7 EDL933. We found that NCCP15739 and NCCP15738 have specific functional genes and pathways related to pathogenicity and motility, and their genomes contained specific prophages. NCCP15739 had more genes (410), virulence factors (48), and phage-related regions (11) than NCCP15738. However, NCCP15738 had three more antibiotic resistance genes than NCCP15739. To investigate the effect of these differences on pathogenicity and biological properties, further studies are needed.

## Additional files

**Additional file 1: Table S1.** Selected genes from NCCP15739 and NCCP15738 for functional annotation clustering.

**Additional file 2: Table S2.** Metabolic pathway comparison of NCCP15739 and NCCP15738.

**Additional file 3: Table S3.** Virulence genes of NCCP15739 and the reference strains.

**Additional file 4: Table S4.** Phage-associated regions in NCCP15739 and the reference strains.

**Additional file 5: Table S5.** Antibiotic resistance genes of NCCP15739 and NCCP15738.

## Abbreviations

CDS: coding DNA sequences
EHEC: enterohemorrhagic *Escherichia coli*
IS: insertion sequences
MLST: multi locus sequence typing
NCCP: National Culture Collection for Pathogens
RAST: Rapid Annotation using Subsystem Technology
STEC: Shiga toxin-producing *Escherichia coli*
str.: strain
substr.: substrain
TTSS: type III secretion system

## References

[1] Karmali MA, Steele BT, Petric M, Lim C (1983). Sporadic cases of haemolytic-uraemic syndrome associated with faecal cytotoxin and cytotoxin-producing *Escherichia coli* in stools. Lancet.

[2] Riley LW, Remis RS, Helgerson SD, McGee HB, Wells JG, Davis BR, Hebert RJ, Olcott ES, Johnson LM, Hargrett NT (1983). Hemorrhagic colitis associated with a rare *Escherichia coli* serotype. N Engl J Med.

[3] Wells JG, Davis BR, Wachsmuth IK, Riley LW, Remis RS, Sokolow R, Morris GK (1983). Laboratory investigation of hemorrhagic colitis outbreaks associated with a rare *Escherichia coli* serotype. J Clin Microbiol.

[4] Nataro JP, Kaper JB (1998). Diarrheagenic *Escherichia coli*. Clin Microbiol Rev.

[5] Caprioli A, Morabito S, Brugere H, Oswald E (2005). Enterohaemorrhagic *Escherichia coli*: emerging issues on virulence and modes of transmission. Vet Res.

[6] Zhang WL, Kohler B, Oswald E, Beutin L, Karch H, Morabito S, Caprioli A, Suerbaum S, Schmidt H (2002). Genetic diversity of intimin genes of attaching and effacing *Escherichia coli* strains. J Clin Microbiol.

[7] Clarke SC, Haigh RD, Freestone PP, Williams PH (2003). Virulence of enteropathogenic *Escherichia coli*, a global pathogen. Clin Microbiol Rev.

[8] Garmendia J, Frankel G, Crepin VF (2005). Enteropathogenic and enterohemorrhagic *Escherichia coli* infections: translocation, translocation, translocation. Infect Immun.

[9] Makino S, Tobe T, Asakura H, Watarai M, Ikeda T, Takeshi K, Sasakawa C (2003). Distribution of the secondary type III secretion system locus found in enterohemorrhagic *Escherichia coli* O157:H7 isolates among Shiga toxin-producing *E. coli* strains. J Clin Microbiol.

[10] Taylor KA, O’Connell CB, Luther PW, Donnenberg MS (1998). The EspB protein of enteropathogenic *Escherichia coli* is targeted to the cytoplasm of infected HeLa cells. Infect Immun.

[11] Blank TE, Lacher DW, Scaletsky IC, Zhong H, Whittam TS, Donnenberg MS (2003). Enteropathogenic *Escherichia coli* O157 strains from Brazil. Emerg Infect Dis.

[12] Kwon T, Kim JB, Bak YS, Yu YB, Kwon KS, Kim W, Cho SH (2016). Draft genome sequence of non-shiga toxin-producing *Escherichia coli* O157 NCCP15738. Gut Pathog.

[13] Kwon T, Cho SH (2015). Draft Genome Sequence of Enterohemorrhagic *Escherichia coli* O157 NCCP15739, Isolated in the Republic of Korea. Genome Announc.

[14] Guinee PA, Agterberg CM, Jansen WH (1972). *Escherichia coli* O antigen typing by means of a mechanized microtechnique. Appl Microbiol.

[15] Li R, Zhu H, Ruan J, Qian W, Fang X, Shi Z, Li Y, Li S, Shan G, Kristiansen K (2010). De novo assembly of human genomes with massively parallel short read sequencing. Genome Res.

[16] Aziz RK, Bartels D, Best AA, DeJongh M, Disz T, Edwards RA, Formsma K, Gerdes S, Glass EM, Kubal M (2008). The RAST Server: rapid annotations using subsystems technology. BMC Genom.

[17] Li L, Stoeckert CJ, Roos DS (2003). OrthoMCL: identification of ortholog groups for eukaryotic genomes. Genome Res.

[18] Fischer S, Brunk BP, Chen F, Gao X, Harb OS, Iodice JB, Shanmugam D, Roos DS, Stoeckert CJ, Jr.: Using OrthoMCL to assign proteins to OrthoMCL-DB groups or to cluster proteomes into new ortholog groups. Current Protocols Bioinformatics, Chapter 6:Unit 6.12.1–19; 2011. doi:10.1002/0471250953.bi0612s35. 10.1002/0471250953.bi0612s35 PMC3196566 21901743

[19] Chen L, Yang J, Yu J, Yao Z, Sun L, Shen Y, Jin Q (2005). VFDB: a reference database for bacterial virulence factors. Nucleic Acids Res.

[20] Zankari E, Hasman H, Cosentino S, Vestergaard M, Rasmussen S, Lund O, Aarestrup FM, Larsen MV (2012). Identification of acquired antimicrobial resistance genes. J Antimicrob Chemother.

[21] Darling AC, Mau B, Blattner FR, Perna NT (2004). Mauve: multiple alignment of conserved genomic sequence with rearrangements. Genome Res.

[22] Krzywinski M, Schein J, Birol I, Connors J, Gascoyne R, Horsman D, Jones SJ, Marra MA (2009). Circos: an information aesthetic for comparative genomics. Genome Res.

[23] Khan NH, Ahsan M, Yoshizawa S, Hosoya S, Yokota A, Kogure K (2008). Multilocus sequence typing and phylogenetic analyses of *Pseudomonas aeruginosa* Isolates from the ocean. Appl Environ Microbiol.

[24] Glaeser SP, Kampfer P (2015). Multilocus sequence analysis (MLSA) in prokaryotic taxonomy. Syst Appl Microbiol.

[25] Wirth T, Falush D, Lan R, Colles F, Mensa P, Wieler LH, Karch H, Reeves PR, Maiden MC, Ochman H (2006). Sex and virulence in *Escherichia coli*: an evolutionary perspective. Mol Microbiol.

[26] Sahl JW, Matalka MN, Rasko DA (2012). Phylomark, a tool to identify conserved phylogenetic markers from whole-genome alignments. Appl Environ Microbiol.

[27] Angiuoli SV, Salzberg SL (2011). Mugsy: fast multiple alignment of closely related whole genomes. Bioinformatics.

[28] Tavaré S (1986). Some probabilistic and statistical problems in the analysis of DNA sequences. Lect Math Life Sci.

[29] Stamatakis A: Phylogenetic models of rate heterogeneity: a high performance computing perspective. In: Parallel and distributed processing symposium, 2006 IPDPS 2006 20th international 2006.

[30] Price MN, Dehal PS, Arkin AP (2009). FastTree: computing large minimum evolution trees with profiles instead of a distance matrix. Mol Biol Evol.

[31] Zhou Y, Liang Y, Lynch KH, Dennis JJ, Wishart DS (2011). PHAST: a fast phage search tool. Nucleic Acids Res.

[32] McCarter LL (2004). Dual flagellar systems enable motility under different circumstances. J Mol Microbiol Biotechnol.

[33] Wackett LP, Sadowsky MJ, Martinez B, Shapir N (2002). Biodegradation of atrazine and related s-triazine compounds: from enzymes to field studies. Appl Microbiol Biotechnol.

[34] Neilands JB (1995). Siderophores: structure and function of microbial iron transport compounds. J Biol Chem.

[35] Franke J, Ishida K, Hertweck C (2014). Evolution of siderophore pathways in human pathogenic bacteria. J Am Chem Soc.

[36] Skaar EP (2010). The battle for iron between bacterial pathogens and their vertebrate hosts. PLoS Pathog.

[37] Figueiredo JC, Grau MV, Haile RW, Sandler RS, Summers RW, Bresalier RS, Burke CA, McKeown-Eyssen GE, Baron JA (2009). Folic acid and risk of prostate cancer: results from a randomized clinical trial. J Natl Cancer Inst.

[38] Ley RE, Hamady M, Lozupone C, Turnbaugh PJ, Ramey RR, Bircher JS, Schlegel ML, Tucker TA, Schrenzel MD, Knight R (2008). Evolution of mammals and their gut microbes. Science.

[39] Teufel R, Mascaraque V, Ismail W, Voss M, Perera J, Eisenreich W, Haehnel W, Fuchs G (2010). Bacterial phenylalanine and phenylacetate catabolic pathway revealed. Proc Natl Acad Sci USA.

[40] Chai Y, Beauregard PB, Vlamakis H, Losick R, Kolter R (2012). Galactose metabolism plays a crucial role in biofilm formation by *Bacillus subtilis*. MBio.

[41] Pallen MJ, Beatson SA, Bailey CM (2005). Bioinformatics analysis of the locus for enterocyte effacement provides novel insights into type-III secretion. BMC Microbiol.

[42] Burgos Y, Beutin L (2010). Common origin of plasmid encoded alpha-hemolysin genes in *Escherichia coli*. BMC Microbiol.

[43] Lim JY, Yoon J, Hovde CJ (2010). A brief overview of *Escherichia coli* O157:H7 and its plasmid O157. J Microbiol Biotechnol.

[44] Lee JE, Reed J, Shields MS, Spiegel KM, Farrell LD, Sheridan PP (2007). Phylogenetic analysis of Shiga toxin 1 and Shiga toxin 2 genes associated with disease outbreaks. BMC Microbiol.

[45] Colomer-Lluch M, Imamovic L, Jofre J, Muniesa M (2011). Bacteriophages carrying antibiotic resistance genes in fecal waste from cattle, pigs, and poultry. Antimicrob Agents Chemother.

[46] O’Brien AD, Newland JW, Miller SF, Holmes RK, Smith HW, Formal SB (1984). Shiga-like toxin-converting phages from *Escherichia coli* strains that cause hemorrhagic colitis or infantile diarrhea. Science.

[47] Ventura M, Canchaya C, Bernini V, Altermann E, Barrangou R, McGrath S, Claesson MJ, Li Y, Leahy S, Walker CD (2006). Comparative genomics and transcriptional analysis of prophages identified in the genomes of *Lactobacillus gasseri*, *Lactobacillus salivarius*, and *Lactobacillus casei*. Appl Environ Microbiol.

